# Clinical outcomes in pediatric hemodialysis patients in the USA: lessons from CMS’ ESRD CPM Project

**DOI:** 10.1007/s00467-008-0831-0

**Published:** 2009-07-01

**Authors:** Alicia M. Neu, Diane L. Frankenfield

**Affiliations:** 1 Division of Pediatric Nephrology, Johns Hopkins Medicine, 200 North Wolfe Street, Room 3065, Baltimore, MD 21287 USA; 2 Office of Research, Development and Information, The Centers for Medicare & Medicaid Services (CMS), Baltimore, MD USA

**Keywords:** Pediatric, Hemodialysis, Outcomes, Clinical performance measures, CPM, Hospitalization, Mortality

## Abstract

Although prospective randomized trials have provided important information and allowed the development of evidence-based guidelines in adult hemodialysis (HD) patients, with approximately 800 prevalent pediatric HD patients in the United States, such studies are difficult to perform in this population. Observational data obtained through the Center for Medicare & Medicaid Services’ (CMS’) End Stage Renal Disease (ESRD) Clinical Performance Measures (CPM) Project have allowed description of the clinical care provided to pediatric HD patients as well as identification of risk factors for failure to reach adult targets for clinical parameters such as hemoglobin, single-pool Kt/V (spKt/V) and serum albumin. In addition, studies linking data from the ESRD CPM Project and the United States Renal Data System have allowed evaluation of associations between achievement of those targets and the outcomes of hospitalization and death. The results of those studies, while unable to prove cause and effect, suggest that the adult ESRD CPM targets may assist in identifying pediatric HD patients at risk for poor outcomes.

## Introduction

**Fig. 1:**
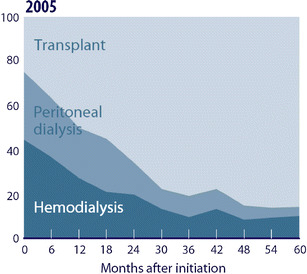
Prevalent patient distribution by modality for pediatric (0–19 years) end-stage renal disease patients for 2005, taken from the 2007 United States Renal Data Systems Annual Report (USRDS) [4]

The gold standard in clinical research is the prospective randomized controlled study and trials such as the National Cooperative Dialysis Study performed in the 1970s and more recently the Hemodialysis (HEMO) Study have provided important information that has supported the development of evidence-based guidelines for the clinical care of adult hemodialysis (HD) patients [1–3]. Such studies are difficult to perform in pediatric HD patients given the limited number of patients and the fact that, because kidney transplantation is the treatment of choice for the majority of pediatric end-stage kidney disease (ESRD) patients, dialysis is often provided as a relatively short-term therapy until transplantation can be performed. Figure 1 shows the prevalent pediatric ESRD population in the United States from 2005 and demonstrates that whereas >40% of patients at ESRD initiation are on HD, the majority of patients who have had ESRD for 24 months or longer have received a kidney transplant [4]. Thus, the pediatric ESRD population is small and fluid, moving from dialysis to kidney transplant to chronic kidney disease and back to dialysis or transplant, making prospective study in patients on any single modality difficult. This limitation is coupled with the fact that the incidences of hard outcomes such as mortality are relatively low when compared with adult ESRD patients [4].

Although large-scale, prospective randomized studies in pediatric HD patients are lacking, there are several observational studies that have provided important information regarding the care provided to pediatric ESRD patients. An example is the Centers for Medicare & Medicaid Services’ (CMS’) ESRD Clinical Performance Measures (CPM) Project. Since 2000, the ESRD CPM Project has collected clinical data on pediatric HD patients and has provided important information about the care provided to pediatric HD patients in the United States [5–12]. In addition, several studies have linked the intermediate outcomes collected by the ESRD CPM Project with administrative data from the USRDS in an effort to demonstrate associations between the attainment of certain clinical targets (such as a hemoglobin ≥ 11 g/dl) and morbidity and mortality [13–15]. Although these studies have added significantly to the information available to those who care for children on HD, it is important to understand the limitations of these data—perhaps most importantly the inability of these studies to demonstrate causality. The purpose of this article is to review data available on pediatric HD patients from the ESRD CPM Project and studies linking data from the ESRD CPM Project with USRDS administrative data. To elucidate the strengths and limitations of these data, the history and structure of the ESRD CPM Project will be reviewed briefly. A detailed history of the project can be found on the CMS Web site (https://doi.org/www.cms.hhs.gov/CPMProject).

## History and background of CMS’ ESRD CPM project

All patients with ESRD in the United States, including children, have been eligible to receive Medicare coverage since 1972. As part of its oversight of the Medicare program, CMS contracts with 18 ESRD network organizations throughout the United States to monitor the quality of care for ESRD patients and to facilitate improvements in that care. Since 1994, this quality assurance effort has included collection of clinical parameters in the areas of anemia management, dialysis adequacy, and serum albumin [5, 16]. In 1997, CMS funded the development of Clinical Performance Measures (CPMs) to be based on the National Kidney Foundation (NKF) Dialysis Outcomes Quality Initiative (DOQI) Guidelines [17–20]. Sixteen ESRD CPMs were developed that have subsequently been reduced to 13: three for HD adequacy, three for peritoneal dialysis (PD) adequacy, three for vascular access, and four for anemia management [5]. A full description of the CPMs is presented on the CMS Web site (https://doi.org/www.cms.hhs.gov/CPMProject) [5]. Similar to the KDOQI guidelines on which they are based, the ESRD CPMs establish targets for the frequency of monitoring the various clinical parameters, as well as thresholds or targets for the parameters themselves. That is, within the anemia-management CPMs, the target level for hemoglobin is specified, as is the frequency of monitoring serum transferrin saturation (TSAT) and serum ferritin and the target values for serum TSAT and serum ferritin [5]. In addition to collecting information necessary to calculate the ESRD CPMs, the project also collects information on other aspects of dialysis care. In particular, although measurement of or obtaining a certain level of serum albumin is not a CPM, these data are felt to be an important indicator of care, so they are collected and reported by the ESRD CPM Project [5]. It should be noted that the ESRD CPMs are for adult dialysis patients, and at present, there are no pediatric ESRD CPMs [5].

**Fig. 2:**
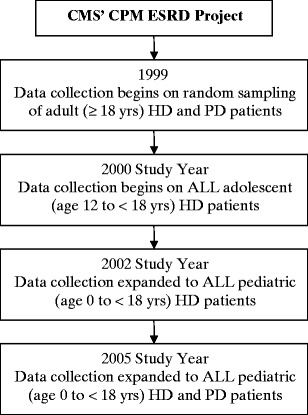
Timeline for expansion of End-Stage Renal Disease Clinical Performance Measures (ESRD CPM) Project to include data collection on pediatric dialysis patients [5]

In 1999 the ongoing quality assurance data collection effort by the networks was merged with the ESRD CPM Project, and since that time, the project has collected data on a random sampling of adult HD and PD patients [5]. The results of these data collection efforts are reported in an annual report (https://doi.org/www.cms.hhs.gov/CPMProject) as well as in more than 50 publications [5]. Data on pediatric dialysis patients were not collected until the 2000 study year. Figure 2 diagrams the timeline of data collection expansion to include pediatric dialysis patients. It should be noted that unlike the random sampling that occurs in adult dialysis patients, data are collected on all pediatric patients on dialysis at the time of the data collection. Furthermore, identifying pediatric dialysis patients for data collection is not restricted to patients who have Medicare as primary payer for their dialysis services. Thus, all pediatric dialysis patients, regardless of insurance coverage, will be identified and have data submitted to the ESRD CPM Project. Because this article focuses on outcomes studies in pediatric HD patients, the remainder of the description of the data collection effort discusses the procedure for patients on that modality.

## Data collection for ESRD CPM Project

The data collection for the ESRD CPM Project occurs in the spring of each study year, when a five-page data collection form is distributed to all facilities that have been identified by the 18 ESRD networks as having a pediatric (age < 18 years) patient receiving in-center HD on December 31 of the preceding year. The dialysis facilities provide clinical information abstracted from patients’ medical records for the months of October, November, and December of the preceding year. That is, for the 2006 CPM study year, data are abstracted from October through December 2005 on any pediatric patient alive and on in-center HD as of 31 December 2005. Demographic information provided by the facility includes gender, race, ethnicity, age, primary cause of ESRD, and date of first dialysis. Information used to assess the dialysis care provided to these patients includes patient height, pre- and postdialysis weight, pre- and postdialysis blood urea nitrogen (BUN) values, dialysis session length, dialyzer KUf values, reported urea reduction ratios and reported Kt/V values, type of vascular access [catheter, arteriovenous fistula (AVF), arteriovenous graft (AVG)], blood-pump flow rates, monitoring of the access site for stenosis, monthly hemoglobin, TSAT, serum ferritin, and serum albumin values [with the associated laboratory method—bromcresol green (BCG) or bromcresol purple (BCP) [21], as these two methods have been shown to yield systematically different results], erythropoietin dose and route of administration, and iron prescription information. Although reported Kt/V values are collected on the data collection form, single-pool Kt/V (spKt/V) values presented in the annual report and used for subsequent analysis are calculated from the raw data points using the Daugirdis II formula [22]. Height, weight, and body mass index (BMI) standard deviation scores (SDS) are calculated using the appropriate gender-/age-specific mean, SD and adjustment parameters for the national population derived from the Third National Health and Nutrition Examination Survey (NHANES III) (2000) of the National Center for Health Statistics [23]. Although data are requested on all prevalent pediatric HD patients, to remain in the analysis, a patient must have the minimum data elements submitted, which include a completed data collection form with at least one monthly hemoglobin value, at least one paired pre- and postdialysis BUN value, and at least one serum albumin value over the 3-month study period [5].

## General limitations and strengths of the ESRD CPM Project

Now that the process of the ESRD CPM Project has been described, before presenting the data from that project, it is important to reiterate some of the key points of the data collection process that highlight the strengths and weaknesses of the data and their applicability to pediatric HD patients in general. First and foremost, data collection occurs on a prevalent, not incident, population. That is, patients are not enrolled at the time of dialysis initiation and followed over time; rather, the ESRD CPM Project provides a snapshot of the care provided over a 3-month period to patients on dialysis at the time of data collection. It cannot be assumed that these 3 months accurately reflect the long-term status of the patient. The second general limitation of the data collection is that many factors that may impact the ability to attain the various threshold values, such as adherence to prescribed oral therapies, adherence to dialysis treatments, markers of inflammation beyond serum albumin and serum ferritin, and nutritional assessment beyond height, weight and serum albumin, are not collected by the ESRD CPM Project and therefore any influence cannot be evaluated or factored. Finally, the ESRD CPM Project focuses on intermediate outcomes of anemia management, vascular access, dialysis adequacy, and serum albumin and does not collect data on the more firm clinical outcomes of neurocognitive development, hospitalization, or mortality. Although data on linear growth, another clinical outcome in pediatric patients, are collected, data on factors that may affect linear growth, such as Tanner stage, calcium and phosphorous balance, and prescription of recombinant human growth hormone, have not been routinely collected [5]. Finally, measures of quality of life are not obtained, although a special analysis to evaluate quality of life in pediatric dialysis patients in several ESRD networks is underway [24].

Although data on hospitalization and mortality are not available in the ESRD CPM Project, they are available in the United States Renal Data System (USRDS) [4]. Thus, by linking the intermediate outcomes in the ESRD CPM Project with hospitalization and mortality data in USRDS, associations between achieving threshold values for the adult ESRD CPMs and hospitalization and mortality can be evaluated. A full description of the USRDS, including details of how to obtain data from the USRDS and ESRD CPM Project for research, is available in a published annual report and on the Internet (https://doi.org/www.usrds.org) [4]. It should be noted that although all ESRD patients in the United should have a file in USRDS, information in the USRDS on hospitalization are obtained from Medicare billing claims. Therefore, only patients for whom a bill was submitted to Medicare will have that hospitalization data captured [4].

The strengths of the ESRD CPM Project data set lie in the fact that it captures information on all prevalent pediatric HD patients, unlike data collection in adult dialysis patients, which occurs on a representative sample. Not only does the ESRD CPM Project allow evaluation of the universe of pediatric HD patients in each study year, but patients who remain on dialysis will have data submitted in sequential study years, allowing longitudinal assessment of clinical parameters in a subgroup of patients. Finally, data for the ESRD CPM Project are collected for monitoring clinical care, and outlying values are verified [5]. This is in contrast to data obtained from the USRDS, for example, where data are abstracted from forms completed for administrative purposes and are not validated or verified [4].

## Intermediate outcomes in pediatric HD patients

As stated previously, the ESRD CPM Project produces an annual report that provides data collected on the prevalent dialysis population for that study year. For the 2006 study year, 803 pediatric patients were identified as being alive and on HD as of 31 December 2005 [5]. Of these, 743 (93%) had the minimum data submitted to be included in the sample [5]. Table 1 shows the clinical parameters, or intermediate outcomes, of this prevalent population of patients. The annual report also demonstrates that the majority of prevalent pediatric HD patients receive their dialysis by way of a catheter (61%) as opposed to an AVF (31%) or AVG (8%) [5]. Although formal statistical analyses are not performed, the annual report shows the percentage of patients achieving the various threshold values and demonstrates, for example, that in the area of urea clearance, a lower percentage of black than white patients (86% vs 90%), a lower percentage of patients on dialysis for less than 6 months versus 6 months or longer (72% vs 92%), and a lower percentage of patients with serum albumin < 3.5/3.2 g/dl (BCG/BCP) than those with mean serum albumin levels higher than these thresholds (82% vs 89%) had a mean spKt/V ≥ 1.2 [5]. Again, statistical analyses are not performed for the annual report. Therefore, data in the annual report describes the prevalent dialysis population and identifies the percentage of patients who achieve the adult ESRD CPMs, but it does not evaluate whether the differences in the percentage of patients achieving targets is statistically significant, nor does it control for factors that might impact the ability to meet thresholds or targets.

**Table 1:** Clinical parameters in 743 pediatric (< 18 years) hemodialysis patients from the 2006 End-Stage Renal Disease Clinical Performance Measures (ESRD CPM) Project

Clinical Parameter	Mean ± Standard Deviation	Percent equal to or greater than Threshold
spKt/V	1.58 ± 0.33	88% ≥ 1.2
Hemoglobin	11.5 ± 1.6 g/dl	68% ≥ 11 g/dl
Transferrin Saturation	29 ± 14%	74% ≥ 20%
Serum Ferritin	471 ± 471 ng/ml	83% ≥ 100 ng/ml
Serum Albumin	3.9 ± 0.5/3.5 ± 0.5 g/dl (BCG/BCP)	80% ≥ 3.5/3.2 g/dl (BCG/BCP)

Data are from October through December 2005 [5]

*spKt/V* single-pool Kt/V as calculated by the Daugirdas II formula,* BCG/BCP* bromcresol green and bromcresol purple laboratory method

Several studies have used the data from the ESRD CPM Project and performed multivariate analysis specifically to delineate risk factors for failing to meet these threshold values [6, 7]. A study by Frankenfield et al. used data from the 2000 study year, which included 433 adolescent (age 12 to < 18 years) HD patients [6]. The results of multivariate logistic regression analysis to identify factors predictive of failing to reach threshold values for spKt/V, serum albumin, and hemoglobin are shown in Table 2 [6]. Perhaps most notably, black patients were significantly less likely to achieve an spKt/V ≥ 1.2 than were white patients, despite controlling for multiple possible confounding variables [6]. Also of note, failure to meet the threshold for either serum albumin or hemoglobin was a risk factor for failing to meet the threshold for the other (Table 2) [6]. A subsequent analysis evaluated data from the 2001 study year, which included 435 patients aged 12 to < 18 years on HD, and focused entirely on risk factors for anemia, defined as hemoglobin < 11 g/dl [7]. In multivariate analysis, factors that remained predictive of anemia in the final model included duration of dialysis less than 6 months [odds ratio (OR) 3.5, 95% confidence interval (CI) 1.9, 6.5], mean serum albumin < 3.5/3.2 g/dl (BCG/BCP) (OR 3.2, 95% CI 1.8, 5.8) and mean TSAT < 20% (OR 1.9, 95% CI 1.2, 3.2) [7].

**Table 2:** Factors remaining predictive of achieving threshold values for single-pool Kt/V (spKt/V), mean hemoglobin, and mean serum albumin in 433 adolescent hemodialysis (HD) patients after multivariate logistic regression analyses

Clinical Parameter	Threshold	Factor	Odds Ratio	95% Confidence Interval
spKt/V	≥ 1.2	Female gender	2.8	1.4, 5.9
		Black race	0.43	0.21, 0.86
		Body surface area (highest quartile = referent)		
		Quartile 1	91.6	23.3, 360.2
		Quartile 2	35.4	11.6, 107.9
		Quartile 3	9.3	3.6, 24.1
		Mean serum albumin ≥ 3.5/3.2 g/dl (BCG/BCP)	3.2	1.4,7.3
		Increasing mean dialysis session length (min)	1.03	1.02,1.05
		Increasing mean blood-pump flow rate (ml/min)	1.016	1.010,1.021
Serum albumin	≥ 3.5/3.2 g/dl (BCG/BCP)	Hispanic ethnicity	2.6	1.1, 5.8
		Duration of dialysis (2 + years = referent)		
		< 0.5 years	0.24	0.12, 0.47
		0.5–0.9 years	0.43	0.20, 0.93
		1.0–1.9 years	NS	
		Increasing mean hemoglobin (g/dl)	1.5	1.3, 1.8
Hemoglobin	≥ 11 g/dl	Lower mean erythropoietin dose (U/kg/dose)	0.990	0.986, 0.994
		Mean serum albumin ≥ 3.5/3.2 g/dl (BCG/BCP)	4.2	2.2, 7.9

Factors entered into the models included age, duration of dialysis, cause of end-stage renal disease, access type, body surface area, gender, increasing hemoglobin, increasing spKt/V, and increasing serum albumin [6]

*BCG/BCP* bromcresol green/bromcresol purple laboratory method

Together, these two studies identify factors that are associated with failure to reach target values for urea clearance, hemoglobin, and serum albumin. However, it must reiterated that data are obtained only from a 3-month period and may not, therefore, be reflective of the typical status of the patient. In fact, an analysis of longitudinal data revealed that among patients who had data submitted to two consecutive study years, a significant percentage of patients who failed to meet threshold values for spKt/V, serum albumin, and hemoglobin in the first study year exceeded those values in the following year [8]. It is also important to reiterate that these data evaluate the likelihood that pediatric HD patients will achieve a threshold value and do not evaluate whether or not achievement of that threshold is, in fact, associated with an improvement in patient outcome.

## Association between achievement of adult ESRD CPMs and clinical outcomes

### Height and linear growth

Several studies have delineated associations between the achievement of the adult targets collected by the ESRD CPM Project and clinical outcomes, specifically height/linear growth, hospitalization, and mortality [8–10, 12–14]. A study by Gorman et al. used data from the 2002 ESRD CPM study year to determine clinical predictors of short stature, defined as a height SDS < −1.88, which corresponds to the third percentile for age and gender [10]. The 2002 study year contained information on 651 pediatric (age < 18 years old) HD patients, of whom 266 (41%) had a height SDS < −1.88 [10]. Multivariate analyses were done separately in patients aged 10 to < 15 years of age and those 15 to < 18 years of age, the results of which are shown in Tables 3 and 4, respectively [10]. The number of patients < 10  years of age was too small (90) to allow statistical analysis in this subgroup [10].

**Table 3:** Adjusted odds ratios of final logistic multivariate model predicting short stature in pediatric hemodialysis patients 10 to < 15 years old

Predictor (123)	Odds Ratio (95% Confidence Interval)	*P* value
Female (vs male)	2.9 (1.1–7.1)	< 0.05
Black (vs white)	3.2 (1.2–9.1)	< 0.05
Hispanic (vs non-Hispanic)	4.5 (1.3–15.3)	< 0.05
Congenital/urologic cause of ESRD (vs acquired/other)	5.4 (2.1–13.8)	< 0.001
Years on dialysis (per 1 year)	1.2 (1.1–1.4)	< 0.01
Increase in nPCR (0.1 g/kg/day)	1.3 (1.1–1.5)	< 0.01

Candidate factors considered for final model and excluded for nonsignificance: spKt/V, albumin < 3.5/3.2 (BCG/BCP), and hemoglobin

*BCG* bromcresol green method,* BCP* bromcresol purple method,* ESRD* end-stage renal disease,* nPCR* normalized protein catabolic rate,* spKt/V* single-pooled Kt/V

Reprinted from [10], with permission

**Table 4:** Adjusted odds ratios of final logistic multivariate model predicting short stature in pediatric hemodialysis patients 15 to < 18 years old

Predictor (222)	Odds Ratio (95% Confidence Interval)	*P* value
Male (vs female)	2.6 (1.3–5.2)	< 0.01
Congenital/urologic cause of ESRD (vs acquired/other)	2.8 (1.5–5.4)	< 0.01
Years on dialysis (per 1 year)	1.2 (1.1–1.4)	< 0.001
Increase in spKt/V (0.1 increase)	1.2 (1.1–1.4)	< 0.001
Decrease in mean hemoglobin (1 g/dl)	1.3 (1.04–1.6)	< 0.05

Candidate factors considered for final model and excluded for nonsignificance: race, ethnicity, albumin < 3.5/3.2 (BCG/BCP), and nPCR

*BCG* bromcresol green method,* BCP* bromcresol purple method,* ESRD* end-stage renal disease,* nPCR* normalized protein catabolic rate,* spKt/V* single-pooled Kt/V

Reprinted from [10], with permission

In a more recent analysis, Gorman and colleagues evaluated linear growth, that is, change in height SDS, in 407 patients who had data submitted to two or more consecutive ESRD CPM study years [12]. In multivariate analysis, younger age, longer duration on dialysis, and increasing normalized protein catabolic rate (nPCR) were associated with a worsening in height SDS over subsequent ESRD CPM study years [–0.20 height SDS/year (95% CI –0.3, –0.1) for < 13 years old vs ≥ 13 years old; –0.03 height SDS/year (95% CI -0.14, -0.08) for duration of dialysis ≥ 6 months vs < 6 months; –0.02 height SDS/year (95% CI –0.04, –0.001) for each 0.1 increase in nPCR] [12]. Conversely, female patients and patients who had lower height SDS in their first year of data entry into the ESRD CPM Project had improvement in their height SDS over time [+0.10 height SDS/year (95% CI 0.02, 0.19) for girls vs boys; +0.03 height SDS/year (95% CI 0.01,0.05) for each 0.5 decrease in height SDS] [12]. Etiology of ESRD, race, ethnicity, and achieving target values for hemoglobin, spKt/V, or serum albumin were not significantly associated with either an improvement or worsening in height SDS [12]. These studies suggest that younger age and longer duration of dialysis are associated both with short stature and poor linear growth, even after controlling for multiple possible confounders [10, 12]. It should be reiterated, however, that many factors that might effect linear growth and which are necessary to interpret relationships between intermediate outcomes and linear growth, such as protein intake, use of recombinant growth hormone, Tanner stage, and bone age, are not collected by the ESRD CPM Project.

### Hospitalization and mortality

To evaluate possible associations of the intermediate outcome data obtained by the ESRD CPM Project with the outcomes of hospitalization and mortality, several studies have linked data from the ESRD CPM Project and the USRDS [13–15]. In one study, data from the 2000 and 2001 ESRD CPM study years were linked with hospitalization data from USRDS through 29 January 2001 and mortality data through 15 October 2001 to evaluate associations between attainment of a spKt/V ≥ 1.2 and risk for hospitalization and mortality [13]. Recall that these ESRD CPM study years included only adolescent (age 12 to < 18 years) HD patients [13]. In this study, spKt/V values were available from the ESRD CPM Project on 613 adolescent HD patients, with repeated measures (i.e. values submitted to sequential study years) in 160 (26%) of those patients [13]. Median follow-up for hospitalizations was 7.4 ± 3.8 months and 10.8 ± 2.9 months for mortality [13].

**Fig. 3:**
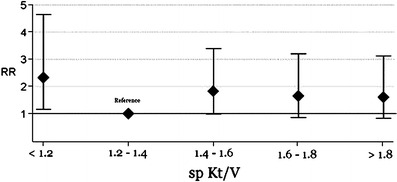
Adjusted relative rate of admission in adolescent (age 12 to < 18 years) hemodialysis patients stratified into five groups by single-pool Kt/V: < 1.2, 1.2–1.4, 1.4–1.6, 1.6–1.8, ≥ 1.8. The group containing values between 1.2 and 1.4 was used as the referent. Factors included in the multivariate analysis: serum albumin, hemoglobin, height standard deviation score, vascular access type, and time since dialysis initiation. Point estimates and 95% confidence interval are shown. Reprinted from [13], with permission

The number of deaths in the cohort was very small at 14, with a mortality rate of 2.0 deaths/100 patient years [13]. The unadjusted hazard rate of death for patients with a spKt/V < 1.2 compared with those with a spKt/V ≥ 1.2 was 2.90 (95% CI 0.75, 9.51) [13]. Given the small number of deaths, multivariate analysis of mortality rates were not performed [13]. Comparison of hospitalization rates revealed that the unadjusted hospitalization rate ratio for spKt/V < 1.2 versus ≥ 1.2 was 1.45 (95% CI 1.00, 2.05) [13]. In multivariate analysis, after adjusting for type of vascular access, serum albumin, hemoglobin, time since dialysis initiation, and presence of short stature, there was no statistically different rate of hospitalization in patients with spKt/V greater than or less than 1.2 [adjusted rate ratio (aRR) 1.59; 95% CI 0.98, 2.56] [13]. However, if the patients were stratified by spKt/V into five subgroups (< 1.2, 1.2 to < 1.4, 1.4 to < 1.6, 1.6–1.8, > 1.8), after adjusting for all the factors listed above, patients with a spKt/V < 1.2 had a significantly higher risk of hospitalization than those with an spKt/V of between 1.2 and 1.4 (aRR 2.46, 95% CI 1.23, 4.94) [13]. These data are shown graphically in the Fig. 3 [13]. Interestingly, increasing spKt/V > 1.4 was not associated with a decreased risk for hospitalization, and in fact, patients with a spKt/V between 1.4 and 1.6 had an increased risk for hospitalization (aRR 1.90, 95% CI 1.02, 3.55) compared with patients with a spKt/V between 1.2 and 1.4 [13].

**Fig. 4:**
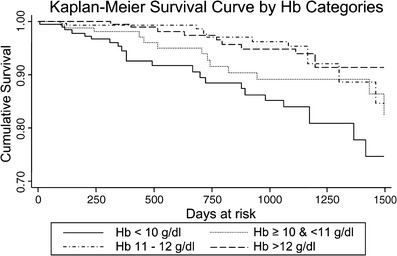
Cumulative survival by hemoglobin categories among adolescent (age 12 to < 18 years) hemodialysis patients. Hemoglobin categories: ^_______^ Hgb < 10 g/dl, --- Hgb ≥ 10% < 11 g/dl, -^.^-^.^-^.^-^.^Hgb 11–12 g/dl, ^_ _ _ _ _^ Hgb > 12 g/dl (*P* = 0.007, log rank). Reprinted from [14], with permission

A study by Amaral and colleagues linked data from the ESRD CPM Project to hospitalization and mortality data from the USRDS to evaluate the relationship between risk for hospitalization and death and the achievement of the adult ESRD CPM minimum target hemoglobin ≥ 11 g/dl [14]. A secondary aim of the study was to assess differential risk for mortality and hospitalization among hemoglobin subcategories: < 10, 10 to < 11, 11 to 12, and > 12 g/dl [14]. This study used data from the 2000 and 2001 ESRD CPM study years and linked these data with hospitalization data from the USRDS through December 2002 and mortality data from the USRDS through November 2003 [14]. The study included data on 677 patients who had data in either the 2000 or 2001 study year and who had hospitalization data submitted to the USRDS. This included 424 patients from the 2000 CPM study year and 430 patients in the 2001 study year [14]. One hundred seventy-seven patients had data submitted to both study years and therefore had repeated measures included in the analysis [14]. Mean follow-up time for hospitalization analysis was 1.7 ± 0.9 years; mean follow-up time for mortality analysis was 2.1 ± 1.3 years [14]. During the follow-up period, 238 (35%) of the 677 patients were hospitalized, and there were 54 deaths [14]. Patients with a hemoglobin < 11 g/dl had a rate of death of 5/100 patient years versus a rate of 1.9/100 patient years in the group with a hemoglobin ≥ 11 g/dl (*p* = 0.009) [14]. In multivariate analysis, hemoglobin ≥ 11 g/dl was associated with a decreased risk for death [hazard ratio (HR) 0.38, 95% CI 0.20, 0.72]. When hemoglobin was evaluated by the subcategories listed above, a significant difference in survival between hemoglobin < 10 g/dl and the other hemoglobin subcategories was demonstrated, and is shown in Fig. 4 (*p* = 0.0007; log rank) [14]. There was a trend toward decreased mortality as hemoglobin increased; hemoglobin 11–12 g/dl was associated with a 69% decreased risk for mortality compared with hemoglobin < 10 g/dl (HR 0.31; 95% CI 0.14, 0.65), and hemoglobin > 12 g/dl was associated with a 72% decreased risk for death compared with hemoglobin < 10 g/dl (HR 0.28, 95% CI 0.13, 0.61) [14].

Unadjusted analysis suggested that a hemoglobin ≥ 11 g/dl was associated with a decreased risk for hospitalization (0.31 ±  0.76 hospitalizations per year at risk vs 0.45 ± 0.87 hospitalizations per year at risk for patients with hemoglobin < 11 g/dl;* p* = 0.0128). However, in multivariate analysis, this was no longer statistically significant (incidence-rate ratio 0.87; 95% CI 0.66, 1.15) [14]. Evaluation of risk for hospitalization by hemoglobin subcategories also did not reveal significant differences [14].

Although these studies demonstrate a strong association between spKt/V and risk for hospitalization and hemoglobin and risk for mortality, respectively, again, these studies determine only associations and not cause and effect. Because the ESRD CPM data were collected after published KDOQI recommendations, it is assumed that values for spKt/V and hemoglobin below the recommended levels were unintentional [13]. Recall, also, that the study of spKt/V evaluated delivered, and not prescribed, dose of dialysis [13]. It is possible that factors that led to reduced delivered dialysis dose, such as shortened runs secondary to noncompliance or medical instability, may have also led to an increased risk for hospitalization [13]. As for the increased risk for hospitalization seen with spKt/V between 1.4 and 1.6, it is also possible that factors that led to the higher spKt/V, either prescribed because of some clinical abnormality such as hyperkalemia or the result of patient factors such as lower volume of distribution of urea (due to lower body weight), also contributed to a higher risk for hospitalization [13]. Similarly, factors that contribute to lower hemoglobin including inflammation, infection, malnutrition, and blood loss might also contribute to the higher mortality rates seen in these patients [14].

## Conclusion

Although mortality and hospitalization rates remain unacceptably high in pediatric HD patients compared with the general pediatric population, death and hospitalization are infrequent events in pediatric HD patients compared with the experience in adult HD patients [4, 25]. With fewer than 800 prevalent United States pediatric HD patients, it is unlikely that meaningful prospective randomized studies evaluating the impact of intermediate outcomes, such as dialysis dose and hemoglobin, on the firmer outcomes of linear growth, hospitalization, and death will ever be conducted in pediatric patients. Therefore, observational studies such as the ESRD CPM Project are important to try to define risk factors for poor outcomes. These studies suggest that the majority of prevalent pediatric HD patients in the United States achieve the threshold values established by the adult ESRD CPMs. However, data from the ESRD CPM Project repeatedly demonstrate that despite controlling for multiple confounders, black race is a significant risk factor for failing to meet the threshold spKt/V of 1.2, a finding that has also been demonstrated in a cohort of pediatric HD patients enrolled in the North American Pediatric Renal Trials and Collaborative Studies [26]. Further study is needed to identify factors responsible for this phenomenon. Patients who fail to meet the threshold value for mean albumin are at risk for failing to reach the threshold value for mean hemoglobin and visa versa, suggesting that these thresholds can be used as markers for patients who are doing poorly. This is emphasized by studies demonstrating that failure to achieve an spKt/V of 1.2 is associated with an increased risk for hospitalization and that failure to achieve a hemoglobin of 11 g/dl is associated with an increased risk of death [13, 14]. Taken together, these data suggest that failure to reach the adult ESRD CPM targets may be a marker of poor outcomes and alert the physician to investigate causes for the failure to achieve them.

## Multiple-Choice Questions

(Answers appear following the reference list)

1. The number of PREVALENT pediatric HD patients in the United States in 2005 was approximately:
  A.) 800
  B.) 5,000
  C.) 1,400
  D.) 12,000
2. Which statement regarding pediatric patients who have had end-stage kidney disease for more than 24 months is correct?
  A.) The majority of patients are treated with HD
  B.) The majority of patients are treated with PD
  C.) The majority of patients have received a kidney transplant
  D.) Roughly equal numbers of patients are treated with dialysis and have received a kidney transplant
3. In pediatric HD patients, which factor has been repeatedly shown to be associated with failing to achieve a single-pool Kt/V of greater than 1.2?
  A.) Male gender
  B.) Focal and segmental glomerulosclerosis as cause of ESRD
  C.) Large body mass index
  D.) Black race
  E.) Hispanic ethnicity
4. Which of the following statements regarding data from CMS’ ESRD CPM Project is true:
  A.) Data are obtained only on patients for whom Medicare is primary payor
  B.) Data are obtained from administrative reports
  C.) Data are obtained from a random sampling of pediatric dialysis patients
  D.) Longitudinal data, or repeated measures, are obtained on pediatric patients who remain on dialysis for two or more consecutive study years
5. Multivariate analyses in pediatric HD patients using data from CMS’ ESRD CPM Project and USRDS have demonstrated which of the following:
  A.) Hemoglobin > 11 g/dl is associated with lower risk for mortality
  B.) Single-pool Kt/V greater than 1.4 is associated with a higher risk for mortality
  C.) Single-pool Kt/V lower than 1.2 is associated with a higher risk of mortality
  D.) Hemoglobin < 11 g/dl is associated with a higher rate of hospitalization
